# Clinical practice variation in orthopedic infections: insights from the Musculoskeletal Infection Society (MSIS) and European Bone and Joint Infection Society (EBJIS) survey, 2023

**DOI:** 10.5194/jbji-10-397-2025

**Published:** 2025-10-28

**Authors:** Laura K. Certain, Irene K. Sigmund

**Affiliations:** 1 Department of Internal Medicine, Division of Infectious Diseases, University of Utah, Salt Lake City, Utah, United States of America; 2 Department of Orthopaedics and Trauma Surgery, Medical University of Vienna, Vienna, Austria

## Abstract

In 2023, members of the Musculoskeletal Infection Society (MSIS) and the European Bone and Joint Infection Society (EBJIS) participated in a survey assessing their approaches to prevention, diagnosis, and management of orthopedic infections. The survey revealed notable differences between the two societies in several key areas, including requirements for smoking cessation prior to elective surgery, use of pre-operative skin and nasal antiseptics, application of local antibiotics in non-infected cases, preferred definitions of periprosthetic joint infection (PJI), use of alpha-defensin testing in pre-operative diagnosis, application of sonication of explanted implants, number of tissue samples obtained for microbiological and histological analysis, use of sequence-based diagnostics, and duration of antibiotic therapy. These findings demonstrate substantial variability in clinical practice among international experts in the field, highlighting the need for further research and consensus to harmonize strategies in orthopedic infection care.

## 1 Introduction

Orthopedic infections are among the most common infections encountered by infectious disease physicians. These conditions, including periprosthetic joint infection (PJI), fracture-related infection (FRI), vertebral osteomyelitis/diskitis (NVO), and diabetic foot infection, pose significant challenges to clinicians. Despite their prevalence, the optimal treatment approach remains debated, with cure rates influenced by both clinical and host-specific factors. Though in recent years we have gained some robust data on management (Li et al., 2019; Bernard et al., 2021; Obremskey et al., 2025), most treatment practices and guidelines are still largely based on expert opinion and retrospective studies.

Two professional societies promoting research and standards within this field are the European Bone and Joint Infection Society (EBJIS) and the Musculoskeletal Infection Society (MSIS, based in the United States). Members of these two societies are global leaders in the field of orthopedic infections, and many have clinical practices that focus almost exclusively on managing these challenging disease entities. While there is often general agreement among these experts on how best to manage a given infection, differences in daily clinical practice remain, likely due to ongoing gaps in the data available.

In 2023, we surveyed attendees of the MSIS annual meeting and members of EBJIS to gather data on current practices within this group of clinicians who focus on orthopedic infections. We present the results here, with the goal of highlighting areas of differing practice that point towards opportunities and needs for future research.

**Table 1:** Comparison of responses from EBJIS and MSIS for questions about pre-operative risk factors. Total N for each question excludes respondents who answered “I don't know” for that question.

	EBJIS		MSIS		
Questions about pre-operative risk factors	N /total	%	N /total	%	p
Delay elective surgery to correct anemia	79/108	73	36/56	64	0.240
Delay elective surgery to correct nutrition	65/110	59	43/58	74	0.053
Hemoglobin A1c above which you do not offer elective surgery					0.014
None	30/93	32	4/42	10	
> 9.0	17/93	18	8/42	19	
> 8.0	19/93	20	17/42	40	
> 7.5	12/93	13	9/42	21	
> 7.0	15/93	16	4/42	10	
Body mass index above which you do not offer elective surgery					0.060
None	52/105	50	20/52	38	
> 50	6/105	6	2/52	4	
> 45	15/105	14	11/52	21	
> 40	24/105	23	16/52	31	
> 35	7/105	7	3/52	6	
> 30	1/105	1	0/52	0	
Usually or always require patients to stop smoking prior to elective surgery	90/117	77	60/65	92	0.009
Offer elective surgery to patients on dialysis	76/112	68	48/60	80	0.091
Routinely screen for *Staphylococcus aureus* colonization pre-op	55/123	45	37/71	52	0.320
Ask all patients to use skin antiseptics at home prior to surgery	49/111	44	53/64	83	<0.001
Use antiseptics in the nares for all patients prior to surgery	34/119	29	34/63	53	<0.001
Use only for patients colonized with *Staphylococcus aureus*	20/119	17	16/63	25	
Use only for patients colonized with methicillin-resistant *S. aureus*	21/119	18	6/63	9	
Use local antibiotics^*^ in non-infected cases (always or sometimes)	40/116	34	29/46	63	<0.001
Use antibiotic cement in non-infected cases (always or sometimes)	92/114	81	41/52	79	0.781

^*^ Local antibiotics = antibiotic powders, calcium sulfate beads, etc.

## 2 Methods

At the MSIS meeting in August 2023, meeting attendees were asked questions using Slido (http://slido.com, last access: 5 August 2023) during the meeting. There were 167 attendees at the meeting, of whom 60 were MSIS members. An email was sent out after the meeting to all MSIS members ( N=246 ) to encourage additional responses and to ask additional questions. EBJIS conducted the survey via email only; an email inviting participation was sent to all EBJIS members ( N=781 ) on 11 December 2023 and was closed on 2 June 2024. Standard summary statistics (means, percentages) were calculated as appropriate. Respondents who selected “I don't know” were excluded from the summary statistics for that question. A chi-squared test was used to determine whether differences between groups were statistically significant.

**Table 2:** Comparison of responses from EBJIS and MSIS for questions about diagnosis of PJI. Total N for each question excludes respondents who answered “I don't know” for that question.

	EBJIS		MSIS		
Questions about PJI diagnostics	N /total	%	N /total	%	p
PJI definition used in clinical practice					<0.001
MSIS 2011 (Parvizi et al., 2011)	5/120	4	29/70	41	
ICM 2013 (Parvizi and Gehrke, 2014)	7/120	6	2/70	3	
ICM 2018/Parvizi 2018 (Shohat et al., 2019; Parvizi et al., 2018)	22/120	18	24/70	34	
EBJIS 2021 (McNally et al., 2021)	74/120	62	6/70	9	
Something else	12/120	10	9/70	12	
In what proportion of suspected PJI cases do you check an alpha-defensin?					<0.001
> 75 %	5/105	5	9	14	
50 %–75 %	6/105	6	6	9	
25 %–49 %	5/105	5	10	15	
10 %–24 %	5/105	5	10	15	
0 %–9 %	84/105	80	31	47	
In what proportion of suspected PJI cases do you send fluid or tissue for sequence-based diagnostics (e.g., PCR, metagenomics)?					0.010
> 75 %	20/111	18	5	8	
50 %–75 %	9/111	8	4	6	
25 %–49 %	14/111	13	7	11	
10 %–24 %	21/111	19	10	15	
0 %–9 %	47/111	42	40	61	
In what proportion of suspected PJI cases do you sonicate explanted hardware?					<0.001
> 75 %	43/113	38	12	18	
50 %–75 %	8/113	7	0	0	
25 %–49 %	0/113	0	1	1	
10 %–24 %	7/113	6	2	3	
0 %–9 %	55/113	49	52	78	
When you suspect infection, how many tissue samples do you take intra-operatively to send for culture?					0.016
5 or more	94/126	75	46/72	64	
3–4	26/126	21	25/72	35	
2	5/126	4	1/72	1	
1	1/126	1	0/72	0	
When you suspect infection, how many tissue samples do you take intra-operatively to send for histology?					0.001
5 or more	29/113	26	8/54	15	
3–4	22/113	19	7/54	13	
2	27/113	24	6/54	11	
1	35/113	31	25/54	46	
0	0/113	0	8/113	15	

## 3 Results

There were 128 respondents to the EBJIS survey (of whom 109 were EBJIS members, 85 %) and 86 respondents to the MSIS survey (53 members, 62 %). A majority of the EBJIS respondents were surgeons (97, 76 %), compared to a minority of the MSIS respondents (23, 27 %). In both groups, over 80 % of respondents were 30–60 years old, though the respondents to the EBJIS survey were further along in clinical practice (61 % in practice > 10 years compared to 43 % of the MSIS respondents). In both groups, about a third of respondents had more than half of their clinical practice devoted to management of orthopedic infection.

**Table 3:** MSIS ID physician members responses to questions about the duration of antibiotic therapy in each clinical scenario.

Minimum antibiotic	*S. aureus* PJI	*S. aureus* NVO	*S. aureus* infection	*S. aureus* infection
treatment	treated with DAIR	with instrumentation	after spinal fusion	after fracture fixation
6 weeks		2 (11 %)	3 (16 %)	4 (21 %)
8 weeks		3 (16 %)	2 (11 %)	
3 months	4 (21 %)	5 (26 %)	3 (16 %)	8 (42 %)
6 months	10 (53 %)	2 (11 %)	3 (16 %)	4 (21 %)
12 months	5 (26 %)	7 (37 %)	8 (42 %)	3 (16 %)

NVO = native vertebral osteomyelitis.

The first set of questions related to patient optimization before elective surgery (Table 1). There are a few notable differences between the two groups. For example, 92 % of MSIS respondents indicated that they required patients to quit smoking prior to elective surgery, compared to only 77 % of EBJIS respondents ( $\chi^{2}$ (1, N=182 ) = 6.825, p=0.009 ). In addition, 83 % of MSIS respondents indicated asking all patients to use skin antiseptics at home prior to surgery, compared to only 44 % of EBJIS respondents ( $\chi^{2}$ (1, N=175 ) = 24.96, p<0.001 ). MSIS respondents were also more likely to indicate the use of topical antibiotics (e.g., powders, calcium sulfate beads) in non-infected cases.

The next set of questions related to diagnosis of PJI (Table 2). Notably, there was little agreement within or between societies on the definition used in clinical practice, with approximately 10 % of respondents from each society using none of the standard PJI definitions. Alpha-defensin was used more frequently among the MSIS respondents, and sequence-based diagnostics were used more frequently by the EBJIS respondents. Use of sonication was bimodal in both groups, presumably due to variability in institutional capabilities. Members of EBJIS reported sending more samples for culture and histology.

There were 19 ID physician members of MSIS who responded to extended survey questions about the duration of antibiotic treatment in various clinical situations: *Staphylococcus aureus* PJI treated with debridement, antibiotics, and implant retention (DAIR); *S. aureus* native vertebral osteomyelitis (NVO) treated with instrumentation; *S. aureus* infection after spinal fusion; *S. aureus* infection after fracture fixation. There was very little agreement among this group of clinicians (Table 3). For example, for patients with *S. aureus* PJI managed with DAIR, 4 treated with systemic antibiotics for a minimum of 3 months, 10 for a minimum of 6 months, and 5 for a minimum of 12 months. Responses were similarly varied for spinal infections and for FRI. Of note, 16 of the 19 respondents reported treating > 100 musculoskeletal infections per year.

## 4 Discussion and conclusion

The survey results presented here provide perspective on current opinions and practices among clinicians specializing in the treatment of musculoskeletal infections. Discrepancies in clinical practice were observed in all areas queried: pre-operative risk management, PJI diagnosis, and treatment duration. These findings underscore substantial variation in clinical practice among experts and point to a clear need for further research to establish evidence-based consensus guidelines.

The responses about pre-operative risk assessment may reflect a difference in cultural approaches to risk management and infection prevention. The MSIS responses suggest a strategy favoring broader preventive measures, even if the absolute benefit is small or unknown; the EBJIS responses suggest more limited interventions. These contrasting philosophies are evident in practices regarding smoking cessation prior to surgery, nasal decolonization, pre-operative skin antisepsis, and the use of local antibiotics in non-infected cases. Such differences may reflect the influence of institutional culture and local interpretation of risk–benefit ratios in shaping clinical routines. The findings of this survey present an opportunity for both EBJIS and MSIS to reflect on the need for a more unified and transparent approach to infection prevention that incorporates both absolute and relative risk.

There was little consensus between and within societies on the definition of PJI used in clinical practice. This diversity is not surprising given the number of different published definitions and the lack of a universally accepted gold standard. Clinicians may default to the one they learned in training or the one most recently endorsed by their affiliated society. Fortunately, a new consensus definition is forthcoming, a result of collaborative effort between EBJIS, MSIS, the Infectious Disease Society of America (IDSA), the International Consensus Meeting (ICM), and the European Society of Clinical Microbiology and Infectious Diseases (ESCMID). The goal is to establish diagnostic criteria that improve accuracy and practicality in clinical practice and enhance comparability across clinical and research settings.

There are of course limitations to data gleaned by voluntary surveys. In addition, due to the make-up of the different societies and the fact that the MSIS survey was conducted in person at the meeting, the comparison is mainly between European surgeons and United States Infectious Disease physicians. The latter group may not be able to assess US surgical practices as accurately as their surgical colleagues. Many people attending the MSIS meeting and responding to the survey are not members of MSIS and may be non-clinicians or trainees who have not yet begun independent practice.

Despite these limitations, these data provide a snapshot of current practices in orthopedic infections across the US and Europe. Looking ahead, stronger collaboration between societies such as EBJIS and MSIS could play a vital role in advancing the field. Joint consensus initiatives, regular international conferences, and collaborative research efforts could help foster mutual understanding of regional practices and priorities. Furthermore, educational programs focusing on the interpretation and application of diagnostic tools may improve the consistency and accuracy of clinical decision-making. Comparative studies evaluating different treatment strategies, including the duration and route of antibiotic therapy, could also help establish clearer, evidence-based standards. Developing shared platforms for data collection and case registries may enhance global benchmarking and facilitate real-world evidence generation. Ultimately, a coordinated international effort can help reduce unwarranted variability in practice and support the development of more cohesive, patient-centered guidelines for the prevention and management of musculoskeletal infections.

## Data Availability

Raw data are available from the authors upon request.

## References

[bib1.bib1] Bernard L, Arvieux C, Brunschweiler B, Touchais S, Ansart S, Bru J-P, Oziol E, Boeri C, Gras G, Druon J, Rosset P, Senneville E, Bentayeb H, Bouhour D, Le Moal G, Michon J, Aumaître H, Forestier E, Laffosse J-M, Begué T, Chirouze C, Dauchy F-A, Devaud E, Martha B, Burgot D, Boutoille D, Stindel E, Dinh A, Bemer P, Giraudeau B, Issartel B, Caille A (2021). Antibiotic Therapy for 6 or 12 Weeks for Prosthetic Joint Infection. N Engl J Med.

[bib1.bib2] Li H-K, Rombach I, Zambellas R, Walker AS, McNally MA, Atkins BL, Lipsky BA, Hughes HC, Bose D, Kümin M, Scarborough C, Matthews PC, Brent AJ, Lomas J, Gundle R, Rogers M, Taylor A, Angus B, Byren I, Berendt AR, Warren S, Fitzgerald FE, Mack DJFF, Hopkins S, Folb J, Reynolds HE, Moore E, Marshall J, Jenkins N, Moran CE, Woodhouse AF, Stafford S, Seaton RA, Vallance C, Hemsley CJ, Bisnauthsing K, Sandoe JATT, Aggarwal I, Ellis SC, Bunn DJ, Sutherland RK, Barlow G, Cooper C, Geue C, Mcmeekin N, Briggs AH, Sendi P, Khatamzas E, Wangrangsimakul T, Wong THHN, Barrett LK, Alvand A, Old CF, Bostock J, Paul J, Cooke G, Thwaites GE, Bejon P, Scarborough M (2019). Oral versus Intravenous Antibiotics for Bone and Joint Infection. N Engl J Med.

[bib1.bib3] McNally M, Sousa R, Wouthuyzen-Bakker M, Chen AF, Soriano A, Vogely HC, Clauss M, Higuera CA, Trebše R (2021). The EBJIS definition of periprosthetic joint infection. Bone Joint J.

[bib1.bib4] Obremskey WT, O'Toole RV, Morshed S, Tornetta P, Murray CK, Jones CB, Scharfstein DO, Taylor TJ, Carlini AR, DeSanto JM, Castillo RC, Bosse MJ, Karunakar MA, Seymour RB, Sims SH, Weinrib DA, Churchill C, Carroll EA, Pilson HT, Goodman JB, Holden MB, Miller AN, Sietsema DL, Stahel PF, Mir H, Schmidt AH, Westberg JR, Mullis B, Shively KD, Hymes RA, Konda SR, Vallier HA, Breslin MA, Smith CS, Crickard CV, Reid JS, Baker M, Eglseder WA, LeBrun C, Manson T, Mascarenhas DC, Nascone J, Pollak AN, Schloss MG, Sciadini MF, Degani Y, Miclau T, Weiss DB, Yarboro SR, McVey ED, Firoozabadi R, Agel J, Burgos EJ, Gajari V, Rodriguez-Buitrago A, Tummuru RR, Trochez KM (2025). Oral vs Intravenous Antibiotics for Fracture-Related Infections. JAMA Surg.

[bib1.bib5] Parvizi J, Gehrke T (2014). Definition of Periprosthetic Joint Infection. J Arthroplasty.

[bib1.bib6] Parvizi J, Zmistowski B, Berbari EF, Bauer TW, Springer BD, Della Valle CJ, Garvin KL, Mont MA, Wongworawat MD, Zalavras CG (2011). New Definition for Periprosthetic Joint Infection: From the Workgroup of the Musculoskeletal Infection Society. Clin Orthop Relat Res.

[bib1.bib7] Parvizi J, Tan TL, Goswami K, Higuera C, Della Valle C, Chen AF, Shohat N (2018). The 2018 Definition of Periprosthetic Hip and Knee Infection: An Evidence-Based and Validated Criteria. J Arthroplasty.

[bib1.bib8] Shohat N, Bauer T, Buttaro M, Budhiparama N, Cashman J, Della Valle CJ, Drago L, Gehrke T, Marcelino Gomes LS, Goswami K, Hailer NP, Han SB, Higuera CA, Inaba Y, Jenny J-Y, Kjaersgaard-Andersen P, Lee M, Llinás A, Malizos K, Mont MA, Jones RM, Parvizi J, Peel T, Rivero-Boschert S, Segreti J, Soriano A, Sousa R, Spangehl M, Tan TL, Tikhilov R, Tuncay I, Winkler H, Witso E, Wouthuyzen-Bakker M, Young S, Zhang X, Zhou Y, Zimmerli W (2019). Hip and Knee Section, What is the Definition of a Periprosthetic Joint Infection (PJI) of the Knee and the Hip? Can the Same Criteria be Used for Both Joints?: Proceedings of International Consensus on Orthopedic Infections. J Arthroplasty.

